# Multi-Type Missing Imputation of Time-Series Power Equipment Monitoring Data Based on Moving Average Filter–Asymmetric Denoising Autoencoder

**DOI:** 10.3390/s23249697

**Published:** 2023-12-08

**Authors:** Ling Jiang, Juping Gu, Xinsong Zhang, Liang Hua, Yueming Cai

**Affiliations:** 1 School of Information Science and Technology, Nantong University, Nantong 226019, China; 2010510006@stmail.ntu.edu.cn; 2 School of Electrical and Information Engineering, Suzhou University of Science and Technology, Suzhou 215101, China; 3 School of Electrical Engineering, Nantong University, Nantong 226019, China; zhang.xs@ntu.edu.cn (X.Z.); hualiang@ntu.edu.cn (L.H.); 4 NARI Technology Company Limited, NARI Group Corporation, Nanjing 211106, China; 13951969176@163.com

**Keywords:** power equipment monitoring data, multi-type missing, data imputation, asymmetric denoising autoencoder, moving average filter

## Abstract

Supervisory control and data acquisition (SCADA) systems are widely utilized in power equipment for condition monitoring. For the collected data, there generally exists a problem—missing data of different types and patterns. This leads to the poor quality and utilization difficulties of the collected data. To address this problem, this paper customizes methodology that combines an asymmetric denoising autoencoder (ADAE) and moving average filter (MAF) to perform accurate missing data imputation. First, convolution and gated recurrent unit (GRU) are applied to the encoder of the ADAE, while the decoder still utilizes the fully connected layers to form an asymmetric network structure. The ADAE extracts the local periodic and temporal features from monitoring data and then decodes the features to realize the imputation of the multi-type missing. On this basis, according to the continuity of power data in the time domain, the MAF is utilized to fuse the prior knowledge of the neighborhood of missing data to secondarily optimize the imputed data. Case studies reveal that the developed method achieves greater accuracy compared to existing models. This paper adopts experiments under different scenarios to justify that the MAF-ADAE method applies to actual power equipment monitoring data imputation.

## 1. Introduction

Nowadays, with the development of sensors and modern communication technology, the supervisory control and data acquisition (SCADA) system in power equipment can realize the real-time acquisition, transmission, and cloud storage of key parameters such as electricity, sound, light, chemistry, and temperature of power equipment. The SCADA system is the backbone used to implement condition-based power equipment maintenance [1,2]. For the SCADA system, the physical parameters of power equipment are mainly collected as structured time-series data. However, in reality, different extents of missing data are a common problem; for example, the long-term strong magnetic environment can cause short-term failures of sensors in substations, and network transmission packet congestion or narrow channel bandwidth variations will cause the loss of long data frames in offshore wind facilities [3]. Missing monitoring data present risks which affect the judgment or prediction of conditions, timely condition maintenance, and the safe and stable operation of the power system [4,5]. Deleting the missing data and data imputation are two classic solutions to address this problem. However, deleting too much data can compromise the accuracy of data-to-characteristics mapping for power equipment. Under such a circumstance, the benefit of data imputation outweighs the method of deleting the missing data. For data imputation, the imputation accuracy stays in high correlation with the data quality. Therefore, a great challenge of the imputation method is that it needs to adapt the characteristics of the missing time-series power equipment monitoring data.

**Figure 1:**
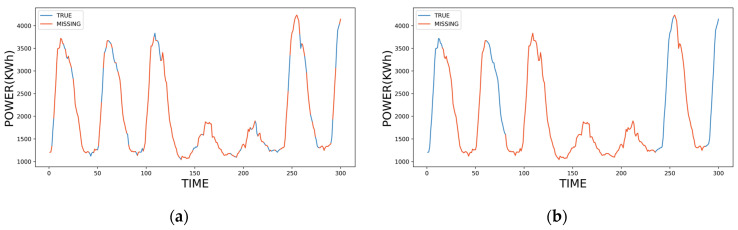
Missing pattern examples of power equipment monitoring data: (**a**) MAR data; (**b**) MNAR data.

For power systems, missing data can be broken down into two types, as shown in Figure 1: Missing at Random (MAR) and Not Missing at Random (MNAR) [6,7]. Figure 1a shows the MAR data in the time-series monitoring data of power equipment, caused by random factors such as random transmission loss or data storage failure. MAR data involve point data loss. In Figure 1b, MNAR data are caused by factors such as data transmission delay or monitoring equipment maintenance. MNAR involves the continuous missing data over a given period.

In the power system industry, the majority of scenarios of missing data are of the MAR type, which has attracted a number of researchers to address it. Existing solutions include the interpolation methods and matrix factorization methods. Among them, interpolation methods, such as the mean interpolation method, Lagrange interpolation method, or Newton interpolation method, were applied to the imputation of MAR data at the earliest stage. Wan et al. [8] applied the mean factorization method to impute the missing values in the power synchronization vector monitoring data. Huang et al. [9] proposed a weighted interpolation data recovery method based on the Lagrange polynomial. However, to control the amount of calculation, the interpolation method used the values near missing points only to construct a polynomial relationship. This characteristic compromises the data imputation performance of reference [9] to the MAR data with a high missing rate. With the mass deployment of SCADA in power equipment, the amount of time-series power data shows exponential growth. It puts forward higher requirements for the accuracy and efficiency of imputation methods. For this reason, related research adopts the matrix factorization method to realize batch imputation according to the low-rank property of the data matrix. Gao et al. [10] proposed a low-rank matrix imputation idea based on singular value thresholding (SVT) and utilized the singular vector of the stored power measurement matrix to restore the MAR data at the next sampling time. The truncated nuclear norm regularization (TNNR) method was utilized in [11] to optimize the approximation of low-rank matrices to impute high-dimensional matrices. This method improved the accuracy of MAR data imputation.

For renewable-dominated power systems, data would be collected and transmitted in a hierarchical manner. This would incur a high delay in data transmission. The segment data that are not transmitted in time will be abandoned, resulting in the frequent occurrence of MNAR in the monitoring data. On top of this problem, a number of studies focus on the imputation of MNAR data. Liao et al. [12] proposed an imputation model based on the alternating direction multiplier method, which can reshape the MNAR data in a single channel of the matrix. However, this method requires a large amount of computation, and is thus hard to apply in online processing. Konstantinopoulos et al. [13] combined adaptive filters on the basis of reference [10] to realize the online data imputation of time-series power data with an improvement in imputation accuracy for MNAR data. Jones et al. [14] transformed the data imputation into a nonlinear constraint problem, solved by using a Kalman filter and smoothing algorithm combined with a quadratic predictor. This method achieved more reliable imputation results. In addition, in a similar scenario to the power equipment monitoring data imputation, Chen et al. [15] proposed a nonconvex, low-rank tensor completion model especially for MNAR data to impute missing spatiotemporal traffic data due to a lack of sensors. The above methods are based on the matrix factorization technique to enhance the imputation performance of MNAR data. However, matrix factorization requires simplifying the system model, causing bias problems in the prediction of the equipment operation data.

In fact, the inherent MAR and the emerging MNAR data coexist in the time-series monitoring data for power equipment. The overall range of data missing rates is also extensive. Previous data imputation models, designed for specific single-type missing data scenarios, cannot be generally applied to address the multi-type and multi-rate scenarios. Some researchers used deep learning methods and carried out imputation research on the multi-type and multi-rate scenarios. Yu et al. [16] utilized a gated recurrent unit (GRU) with a stronger feature extraction ability to form a deep regression neural network to predict the missing data. To estimate missing values in building electric load data, Jeong et al. [17] proposed a data-driven method that incorporates mixture factor analysis to realize multi-type missing imputation. Similarly, Jung et al. [18] used a bagging ensemble of multilayer perceptrons for missing load data imputation. Compared to existing methods, it can effectively solve long-term missing data problems and reduce dependence on historical data. However, the above supervised regression methods require multi-step recursive prediction to realize the imputation of MNAR data. Imputation errors would gradually accumulate with the increase in the number of steps. In [19,20,21], they adopted an unsupervised learning method to achieve the imputation of missing data by mining the spatiotemporal correlation of the data itself. In [19], the generative adversarial network (GAN) was utilized to learn the spatiotemporal feature from power equipment monitoring data. Two groups of networks were driven by a large amount of MAR data to reconstruct the data. Dai et al. [20] compared the missing data to the noise in the data and adopted a lightweight stacked denoising autoencoder (SDAE) to develop an imputation model. They provided a new idea for the imputation of multi-type missing data. On this basis, Li et al. [21] proposed a feature-fusion-based data-restoring model. It utilized a denoising autoencoder (DAE) to impute multi-type missing data collected from a battery management system. Compared with other methods, this method could significantly improve the quality of collected battery operation data. The above unsupervised methods, especially the DAE, do not require data labels and have strong imputation capabilities for multi-type missing data. The DAE is an ideal time-series power data imputation method. In summary, existing research justified that the unsupervised deep learning method is an effective imputation solution for power system data imputation. However, there is a lack of research focusing on data imputation under multiple missing types and multiple missing rates.

In response to the research gap, this paper developed a time-series power equipment monitoring data imputation method combining an asymmetric denoising autoencoder and a moving average filter (MAF-ADAE). The ADAE is capable of learning local periodic and temporal features from the time-series data and utilizes them to impute missing data. Then, a secondary optimization method based on MAF is applied to fuse the neighborhood information near the missing data’s position. The developed MAF-ADAE is expected to capture both periodic and temporal representations of the power equipment monitoring data to realize the imputation of multi-type missing data. The main contribution of this paper is developing a deep learning model combining ADAE and MAF. This two-stage method fuses multiple features to realize the one-time imputation of multi-type missing data in power equipment monitoring data.

The rest of the paper is organized as follows. Section 2 formulates the imputation problem and proposes the ADAE. Section 3 presents MAF to implement the secondary optimization. The experimental setup, including datasets and a benchmark model, as well as the analysis of experimental results and discussion of each module function, is described in Section 4. Section 5 concludes the paper.

## 2. Multi-Type Missing Data Imputation by the ADAE

To achieve data imputation of the multi-type missing for power equipment, first, this section formulates data imputation as a denoising problem and utilizes denoising methods to accomplish imputation. Second, this section proposes an improved ADAE with asymmetric structure, i.e., an encoder that fuses convolution and GRU, a fully connected decoder, and skip connections. Lastly, the improved cost function for training is applied.

### 2.1. Problem Formulation

The MAR and MNAR data in the time-series monitoring data of power equipment are usually set as 0 or nan. They can be essentially equivalent to the white noise commonly existing in continuous data. Therefore, as a general denoising method, denoising autoencoder (DAE) is suitable for the missing imputation task in the time-series data and can recover the missing value to a certain extent. The DAE is an unsupervised deep learning method with noise reduction performance that can effectively remove noise in the data [22]. It has a network structure of encoder–decoder. The encoder in the network acquires the noisy data *x*′ = {*x*′_1_, *x*′_2_, *x*′_3_… *x*′*_n_*} from the given input space *X*′, and extracts the feature *y* to complete the mapping *f* from the input space to the feature space *Y*. The decoder recovers the denoised data *z =* {*z*_1_, *z*_2_, *z*_3_… *z_n_*} according to the feature, and completes the mapping *g* from the feature space to the output space *Z*.

(1) $$\begin{array}{l} f:x'\to y(X'\in x',Y\in y)\\ g:y\to z\;(Z\in z) \end{array}$$

Before applying the DAE, it is necessary to learn a large amount of data and adjust the parameters through cyclic iteration in order to achieve effective mapping. The training process usually reversely acquires the monitoring data *x* from the non-noisy dataset *X*, and manually adds the noise *N* to simulate the noisy data *x*′ monitored in the noise scene for training the model.

(2) x'=x+N

Finally, the DAE is required to optimize the parameters in mapping *f* and *g* to minimize the error between the reconstruction data *z* and the real data *x* to aim the denoising task, as shown in (3).

(3) $$f,g=\arg\underset{f,g}{\min}\left\|X-Z\right\|=\arg\underset{f,g}{\min}\left\|X-g[f(X')]\right\|$$

Corresponding to the missing data in this paper, the obtained value can somewhat be equivalent to noise *N*. Moreover, the missing data are a mixture of MAR and MNAR data, and the differences in the missing rate are significant. Specifically, MAR data in the monitoring data are shown as scattered white noise, MNAR data are shown as continuous white noise in the time domain, and the missing rate covers the range of 0~60%. The continuous long segment missing and highly frequent different types missing in the data mask some key features of the data and make extracting effective features greatly challenging. Therefore, the subsequent work focused on the extraction of periodic and temporal features and fused them to complete the accurate imputation of data.

### 2.2. Asymmetric Denoising Autoencoder

**Figure 2:**
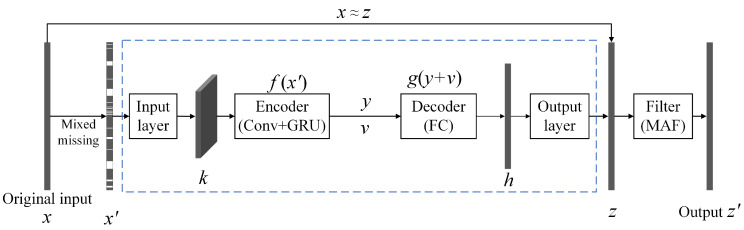
The overall flow of the MAF-ADAE.

Given that the DAE only applies to one-dimensional time-series data, it only cares about changes occurring with time. Thus, it is difficult to extract the long-term dependencies and periodic characteristics of the data, resulting in poor the imputation accuracy of long-lasting missing data. To accommodate the multiple missing types in time-series data with different missing rates, we developed an improved ADAE. The specific structure is shown in the blue dashed box in Figure 2. It consists of an input layer, a hidden layer, and an output layer. The input layer cuts the one-dimensional noisy data *x*′ according to the data collection frequency and stacks it to obtain a two-dimensional square matrix. Then, a single-layer convolution resizes the square matrix to the input matrix *k* and sends it to the hidden layer. The hidden layer includes an encoder and a decoder. The encoder is composed of multiple convolution modules and a GRU module to extract features *y*. The decoder is composed of multiple fully connected layers in series. Furthermore, the global average pooling is adopted to construct skip connections between the encoder and the decoder for transferring low-dimensional features *v*. The output layer adopts a fully connected layer combined with a sigmoid activation function to adjust the output *h* into a one-dimensional matrix consistent with the size of *x*.

**Figure 3:**
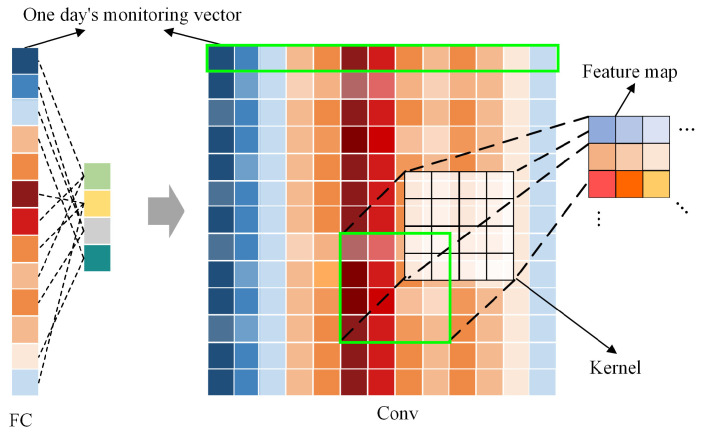
The full connection and convolution for time-series vectors. The different colors represent values of the monitoring data, and dotted green box represents the range of feature extraction.

For the power system industry, the data is usually collected in real time at a resolution of seconds, minutes, or hours. The continuity of the monitoring data in the time domain and the periodicity or regularity of the data exhibited over one or several days can all provide a scientific basis for the missing data imputation. In order to effectively obtain the above characteristics in the data, this paper divides the monitoring data into multiple vectors at intervals of half days or days, and then reorganizes them into matrices, as shown in Figure 3. In this way, the internal variation law of the time-series vectors and the correlation between the vectors can be more intuitively displayed, which provides convenience for feature extraction.

In the encoder, Figure 3 shows the difference in extracting data features via full connection and convolution, respectively. The full connection can only extract features from a single vector, resulting in the weak data mining ability of periodic features. At the same time, with the increasing length of the vector, the number of nodes or layers of the fully connected layer increases. This leads to excessive network parameters, the disappearance of gradients, and increased difficulty in feature extraction. However, convolution as shown in Equation (4) utilizes kernels with different sizes to convolve the matrixes composed of multiple vectors and share the channel weights in the kernel [23]. After multiple layers of convolution, the periodic features in the matrix can be efficiently fused, resulting in a higher-quality feature matrix *y*.

(4) $$r_{i}=f'(r_{i-1})=f_{Rule}(Conv(r_{i-1}))=f_{Relu}(\sum_{j\in M}W_{i}^{j}\times r_{i-1}^{j}+b_{i})$$

where *f*′ represents a layer in the encoder. It has an activation function *f_Relu_* and the convolution layer (*Conv*). *i* is the number of the convolutional layer. *M* is the input feature subset of *r_i_*_−1_, *W_i_^j^* is the convolution kernel, *b_i_* is the bias matrix, and × is the convolution operator.

**Figure 4:**
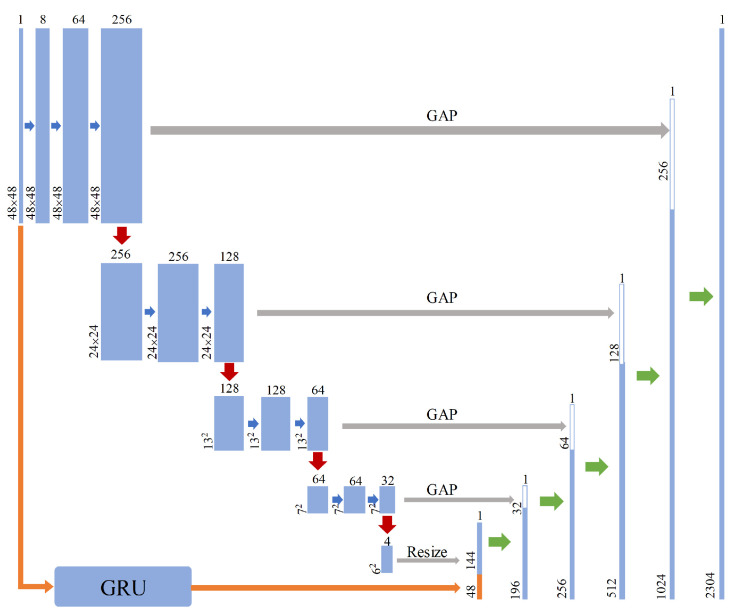
Structure of the ADAE.

Compared with the full connection, the rectangular convolution kernels can fuse the neighborhood vector information around the missing data’s position. Therefore, the convolution can efficiently and deeply extract the high-dimensional periodic features from the matrixes by applying convolution kernels with the multi-scale receptive field. In addition, the convolution only needs to cyclically iterate fewer weight matrices in the convolution kernel. This also enhances the training and testing speed of the model. In Figure 4, the blue arrows represent the convolution with a step size of 1 using a 1 × 1 kernel, without reducing the size of the feature map and only increasing the number of channels. The continuous convolution with 1 × 1 kernel in the feature maps of 48 × 48, 24 × 24, 13 × 13, and 7 × 7 can fuse features between different channels by using only a few parameters. The red arrows represent the convolution with a step size of 2 using a 4 × 4 or 2 × 2 kernel. This could halve the channels for the feature map without changing the size of the feature map. With the decrease in the feature map size, the larger size convolution kernel can effectively reduce the noise caused by missing data and refine high-dimensional features. Finally, the high-dimensional feature matrix of 4 × 6 × 6 is extracted through multi-layer convolution.

Furthermore, in order to extract temporal features in the data, we introduce GRU in the encoder, as shown in the orange arrows in Figure 4 [24]. The GRU is an improved variant of the recurrent neural network (RNN), which inherits a special gated recurrent network structure from the long short-term memory (LSTM) model. The GRU has two gates: update gate *R_t_* and reset gate *Z_t_*. The update gate controls the extent and retains the impact of previous information on the current state. The reset gate determines how to combine new input information with previous memories. The above network structure enables efficient capture of long-term dependencies of time-series data and generates temporal features for decoder data reconstruction. The specific computation of GRU can be given as:

(5) $$\begin{array}{l} Z_{t}=\sigma (W_{z}\cdot [H_{t-1},x_{t}])\\ R_{t}=\sigma (W_{r}\cdot [H_{t-1},x_{t}])\\ A_{t}=\tanh(W_{h}\cdot [R_{t}\times H_{t-1},x_{t}])\\ H_{t}=(1-Z_{t})\times H_{t-1}+Z_{t}*A_{t} \end{array}$$

where *x_t_* is the input vector and *H_t_* is the outcome at time *t. A_t_* is the candidate activation.

In summary, the convolution in the encoder is utilized to extract periodic features from time-series monitoring data, while the GRU is employed for capturing temporal features. Subsequently, the resized one-dimensional vectors formed by concatenating 144 × 1 periodic feature and 48 × 1 temporal feature are treated as input data for decoder data reconstruction.

A greater number of convolutional layers implies the encoder could handle more high-dimensional features but has a higher potential to neglect low-dimensional features. To promote the generality in handling features of different dimensions, this paper adopts the global average pooling (GAP) as skip connections. These skip connections are set before reducing feature map sizes in the convolution, as the represented by the grey arrows in Figure 4. The GAP obtains the low-dimensional feature *v_i_* with *m* × 1 × 1 size by calculating the average value of each channel from the feature map *r_i_* of *m* × *q* × *p* with Equation (5) [25]. The GAP has no parameters that need to be optimized, thus minimizing the overfitting risks of the model. Further, adding the GAP into the encoder could keep the low-dimensional features from the feature maps of different scales maximally to ensure sufficient features for the decoder layer.

(6) $$v_{i}^{m}=s(r_{i}^{m})=\frac{1}{\left|R_{pq}^{m}\right|}\sum_{(p,q)\in R_{pq}}r_{i}^{m,q,p}$$

where *s* represents the GAP in skip connections, *r_i_^m^* is the output of the specified *i*-th convolutional layer, and *R_qp_^m^* is the total number of elements in the feature map with *q* × *p* in the *m*-th channel.

The decoder usually adopts a structure symmetrical to the encoder in the common autoencoder to meet the requirements of input and output dimensions. For example, the encoder and decoder of the DAE all build with fully connected layers. On this basis, the improved convolutional denoising autoencoder (CDAE) adopts convolution and transposed convolution to construct the encoder and decoder, respectively. On the contrary, the transposed convolution is not suitable in the ADAE. This is because the uneven overlap will increase the pixel value at a specific position and reduce the continuity between pixels, resulting in checkboard artefacts in the reconstructed picture [26]. It is acceptable in image reconstruction and will not affect the target information in larger-size images. However, if the transpose convolution is used in the decoder, and then converts the output matrix into time-series data, it will show jagged fluctuations in the time domain. This fluctuation is similar to the high-frequency noise in the signal, and is closely related to the kernel size and step size of the transpose convolution. This continuous abnormal fluctuation does not accord with the operation characteristics of power equipment, and will seriously affect the imputation accuracy of power equipment monitoring data, causing transposed convolution not applicable to this scenario. In summary, to keep the continuity of the reconstructed data, the decoder of the ADAE is still constructed with multiple fully connected layers, as shown by the green arrows in Figure 4. The decoder gradually expands the vectors resized from the high-dimensional feature matrix to reconstruct data. In the process of vector expansion, low-dimensional feature matrixes *v* with a size of 7 × 1 × 1, 13 × 1 × 1, 24 × 1 × 1, and 48 × 1 × 1 are sequentially integrated into the input of the corresponding fully connected layer output *h* to expand the feature information. The decoder of the ADAE is expressed as

(7) hi=g'(hi−1,vi)=fRule(FC(hi−1,vi))=fRule(Wi'[hi−1,vi]+bi')

where *g*′ represents a layer in the decoder. It has an activation function *f_Relu_* and the fully connected layers (*FC*). *i* is the number of the fully connected layer.

### 2.3. Improved Loss Function

The mean square error loss (MSELoss) is commonly used in evaluating the autoencoder. It measures the distance between the true value and the predicted value by calculating the squared error. However, the size of the time-series power equipment monitoring data is usually large. This leads to a high appearance of error of 1 and above in the early training stage. The square operation of MSELoss further amplifies the error, making the convergence of the model difficult. Moreover, the cost function of the end-to-end model usually calculates the loss of the whole data, including missing values and non-missing values, resulting in insufficient attention to the missing. To further improve the optimization performance of the cost function in the ADAE, we propose an improved cost function, which consists of two parts, *L*_1_ and *L*_2_. The *L*_1_ part utilizes a log–cosh loss function to replace the MSELoss to calculate the loss of the whole data.

(8) $$L_{1}(x,z)=\sum_{i=1}^{n}\log(\cosh(e_{i}))=\sum_{i=1}^{n}\log(\cosh(z_{i}-x_{i}))$$

where the *e* is the error. When the *e* is small, the value of the cost function is approximately equal to 0.5 *e*^2^, and the value of the cost function is approximately equal to |*e*| − log2 when the *e* increases. Thus, the error will not be amplified, and the function can perform second-order differentiation everywhere to accelerate the model convergence.

The *L*_2_ part focuses on the missing part in the data. The mean absolute error loss (MAELoss) is utilized in the *L*_2_ part to measure the error of missing which is expressed as the following:

(9) $$L_{2}(x,z)=\sum_{j\in Q}\mathrm{MAELoss}(e_{j})=\sum_{j\in Q}\left|z_{j}-x_{j}\right|$$

where *Q* is a set of missing data’s positions. As shown in (9), the *L*_2_ part only calculates the error of missing. With the error amplification of the MAELoss, the ADAE can pay more attention to missing data imputation. Moreover, the missing only occupies a small part of the data, and the *L*_2_ part will not have a greater impact on the convergence of the model. Ultimately, the loss function of the ADAE is the sum of the *L*_1_ and *L*_2_ parts, which focuses on both the missing part and the overall running trend of the data.

## 3. Imputed Data Optimization by the MAF

**Figure 5:**
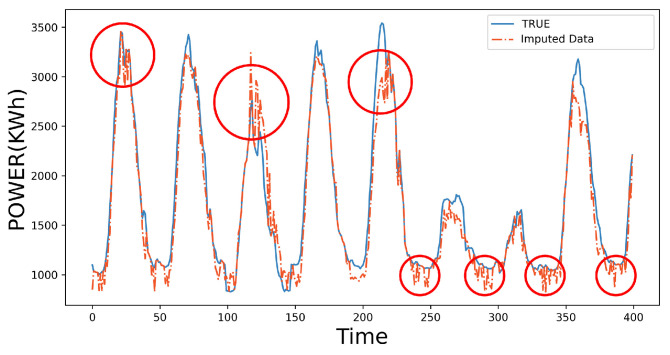
Data imputed by the ADAE under 50% missing rate. The red circles outline the high-frequency fluctuations in the imputed data.

The above ADAE with the end-to-end network can impute the missing part of the input data. The decoder outputs the data with the same length as the input. However, in the real application, missing data are of different types, ratios, and positions. It is vital to find the corresponding value from the output data according to the missing data’s positions in the initial data and then embed the found value into the initial data, in turn, to obtain the complete imputed data as shown in Figure 5.

When the missing rate of data continuously increases, it becomes more difficult for the ADAE to adjust the outputs under a changing position of the missing data. In this case, the lack of sufficient a priori knowledge will cause large deviations in the imputed values. Because of this, the imputed data perform high-frequency fluctuation caused by the alternating occurrence of the imputed value and the non-missing value, as shown in the red circle in Figure 5. To restrict high-frequency fluctuations in the imputed data, this section adopts MAF [27] to fuse imputed values and non-missing values. This stabilizes data fluctuations based on prior knowledge of non-missing values and further optimizes imputed data. The MAF is widely used in signal processing. It is achieved by windowing the time-series signal and obtaining the average value in the window to retain low-frequency effective signals and suppress the high-frequency noises. The filtering process is given as follows:

(10) $$z'(n)=\frac{1}{k}\sum_{i\in [0,k]}z(i-\frac{k-1}{2}+n)$$

where the *n* is the serial number of the missing data, *z*(*n*) is the imputed data by the ADAE, and *z*′(*n*) is the optimized data. *k* is the window length and usually takes an odd value to form a symmetrical window to prevent phase deviation.

**Figure 6:**
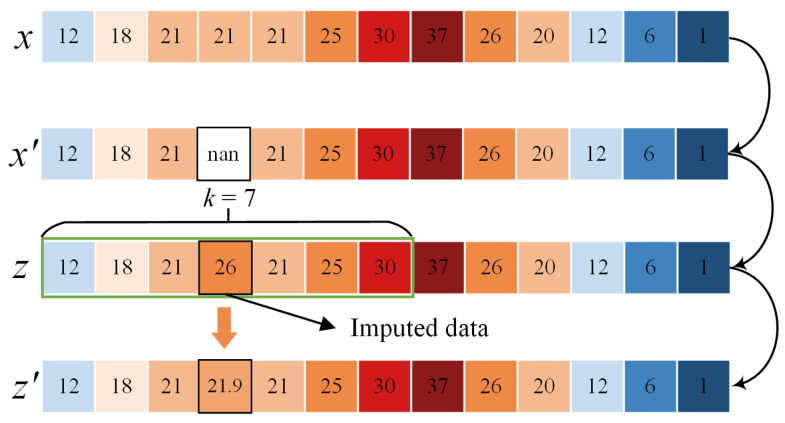
The MAF process for imputed data. The different colors represent values of the monitoring data, and the arrows represent different steps of imputation.

The MAF in this section only needs to fuse the information in the front and back segments of the missing point to optimize the value, so it only needs to add a window at the missing point and calculate the mean value in the window. For example, in Figure 6, the value of the missing point imputed by the ADAE is 26. It does not adhere to the continuity of time-series power equipment data. We perform the MAF with a window length of 7 on the missing point, and the optimized interpolation value is 21.9, which is closer to the real monitoring value of 21.

## 4. Case Studies

This section introduces the case studies for the multi-type missing imputation under three public datasets, including the dataset description, imputation results, repeat tests, and ablation experiments.

### 4.1. Datasets Description

Among the numerous items of monitoring data of power equipment, load data are deemed the most crucial. Thus, we selected three examples of public data to evaluate the developed method: Cardiff conference building energy load data [28], Northern Ireland power energy system load data [29], and Australian electricity load data [30]. These basic data are complete time-series data collected from power equipment. To prepare the data with the missing, we add the multi-type missing of different missing rates to the above three basic data to form new datasets, which are abbreviated as C, N, and A, respectively. Each dataset includes a training set and a validation set. The generation of the training set follows the three steps:

1. Randomly generate the MAR’s rate and the MNAR’s rate within the range of 0~30% to ensure that the total missing rate does not exceed 60%;
2. According to the missing rate, randomly generate a corresponding number of missing locations in the underlying data, and set the value of the position to 0 to form missing data;
3. Repeat steps (1) and (2) 192 times to generate 192 groups of multi-type missing data with different missing rates to form the training set.

The validation set is generated under the missing rate of 30%, 40%, 50%, and 60% of the basic data. The MAR and MNAR scenarios each account for half of the created missing data. The training set and validation set follow the same structure: “group × day × time of day”. A brief description of the three datasets is given as follows:

1. Cardiff conference building energy consumption data (C). They contain power consumption data (KWh) of more than 300 buildings at the Cardiff Conference from 2014 to 2016 in 30 min intervals. The training set contains data from 712 days in 2014–2015. The validation set contains data from 365 days in 2016. The training set size is 192 × 712 × 48. The validation set size is 4 × 365 × 48;
2. Northern Ireland power energy system data (N). They contain electricity demand (MWh) at 15 min intervals in part of Northern Ireland from 2016 to 2018. The training set contains data from 480 days in 2016–2017. The validation set contains data from 240 days in 2018–2019. The training set size is 192 × 480 × 96. The validation set size is 4 × 240 × 96;
3. Australian power load data (A). They contain the electricity demands (MWh) in part of Australia at 30 min intervals from 2006 to 2011. The training set contains data from 1460 days in 2006–2010. The validation set contains data from 365 days in 2010–2011. The training set size is 192 × 1460 × 48. The validation set size is 4 × 365 × 48.

### 4.2. Imputation Results and Accuracy

To justify the imputation performance of the developed ADAE method, we compare it with the multiple effective baseline models selected from the commonly utilized interpolation methods, matrix decomposition methods, and the DAE methods. The baseline models are Lagrange interpolation (LI) [8], temporal regularization matrix factorization (TRMF) [31], low-rank tensor completion truncated kernel norm (LRTC-TNN) [14], SDAE [20], and Variational Autoencoder (VAE) [32]. The mean absolute percentage error (MAPE) and the root mean squared error (RMSE) are selected to measure the imputation performance. These methods are typical evaluation indicators for measuring imputation error, as given by (11) and (12). In (11) and (12), *z*′ is the imputed data in the missing data’s position, and *x* is the corresponding ground truth.

(11) $$\mathrm{MAPE}=\frac{1}{n}\sum_{i=1}^{n}\left|\frac{x_{i}-z{'}_{i}}{x_{i}}\right|\times 100$$

(12) $$\mathrm{RMSE}=\sqrt{\frac{1}{n}\sum_{i=1}^{n}{\left(x_{i}-z{'}_{i}\right)}^{2}}$$

The average MAPE and RMSE of each model over 10 experiment repeats in the three validation sets are shown in Table 1, where the best results are highlighted in bold fonts. In general, the imputation accuracy of the ADAE is better than that of the baseline models in the majority of cases. With the increase in missing rates, the imputation error is exacerbated. The accuracy of the interpolation method represented by LI decreases most significantly. On the contrary, the matrix decomposition methods represented by the TRMF and LRTC-TNN, especially LRTC-TNN, have a low MAPE for the scenario of low missing rates. However, to maintain satisfying imputation performance, a set of key parameters of matrix decomposition methods are required to be fine-tuned. Particularly, once the missing rate or missing type changes, the parameters need to be re-determined. This compromises the application generality in practice. Compared with the above methods, AE methods simulate the actual data missing in the training set, so that they can obtain relatively stable and superior imputation accuracy under different missing rates. On the other hand, since the multi-type missing in time-series power equipment monitoring data does not follow a specific distribution, the MAPE/RMSE of the ADAE proposed in this paper is better than the existing SDAE and VAE. In particular, when the total missing rate exceeds 50%, it still guarantees high imputation accuracy.

**Figure 7:**
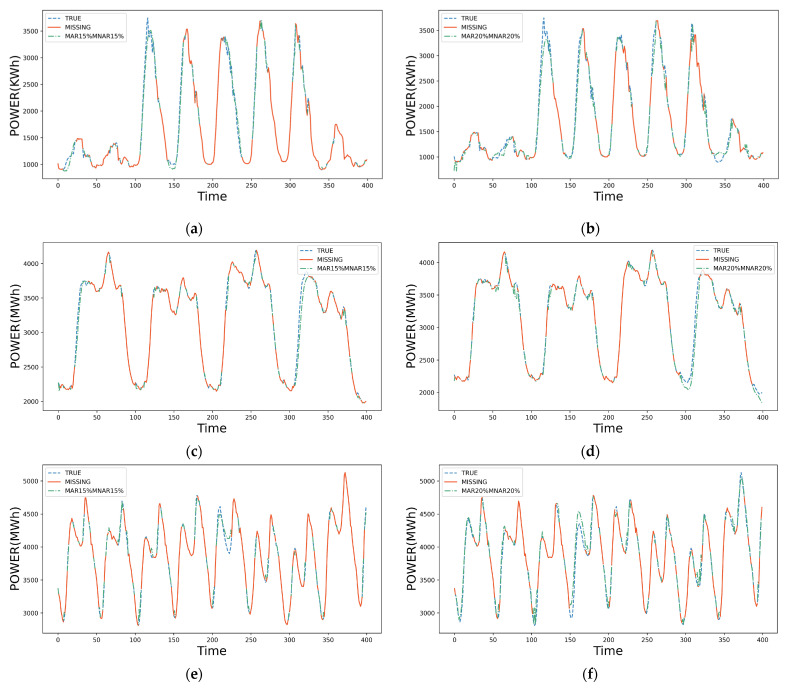
Result of imputation examples with (**a**,**b**) corresponding to dataset C, (**c**,**d**) corresponding to dataset N, and (**e**,**f**) corresponding to dataset A.

In the industry, the data missing rate is usually between 20% and 40%. Therefore, we select two scenarios, where (1) the MAR and MNAR are 15%; and (2) the MAR and MNAR are 20%. The imputation results are shown in Figure 7a–f, where the blue line denotes the true value of monitoring data, the green line denotes the imputed value from MAF-ADAE, and the orange line denotes the monitoring data with multi-type missing. Figure 7 demonstrates that MAF-ADAE can achieve high imputation accuracy for all three datasets, including the scenario in which data change drastically.

The missing data’s position in validation sets is all generated following the missing rate of the MAR and MNAR randomly. Therefore, even with the same missing rate, the test data generated each time are different. To avoid specificity in the single generation of missing data, we further repeated the generation of missing data 10 times at the missing rate of MAR20% MNAR20% and MAR30% MNAR30%. Repeat experiments were carried out in the best-performing models to separately verify the imputation stability in common and extreme missing data scenarios. The standard deviation (SD) of MAPE and RMSE of the results are shown in Table 2, where the best results are marked in bold.

Table 2 shows that the MAF-ADAE achieves the best results in most cases, delivering the greatest imputation accuracy and generalization ability. Moreover, even in extreme missing data scenarios with a missing rate exceeding 50%, MAF-ADAE exhibits strong stability, surpassing the performance of existing methods. It is worth noting that compared with DAE and VAE, which are both autoencoders, the SD of the MAF-ADAE is significantly improved, and the imputation accuracy is more stable. These results demonstrate that the MAF-ADAE can effectively balance the accuracy and stability of the imputation.

### 4.3. Ablation Experiment

**Figure 8:**
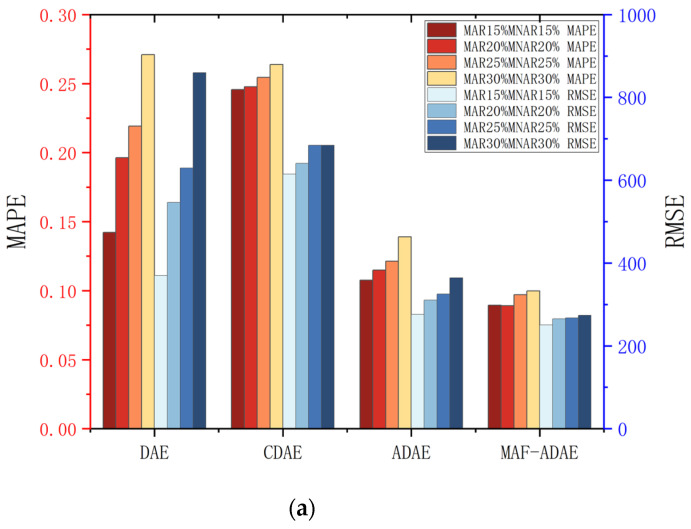

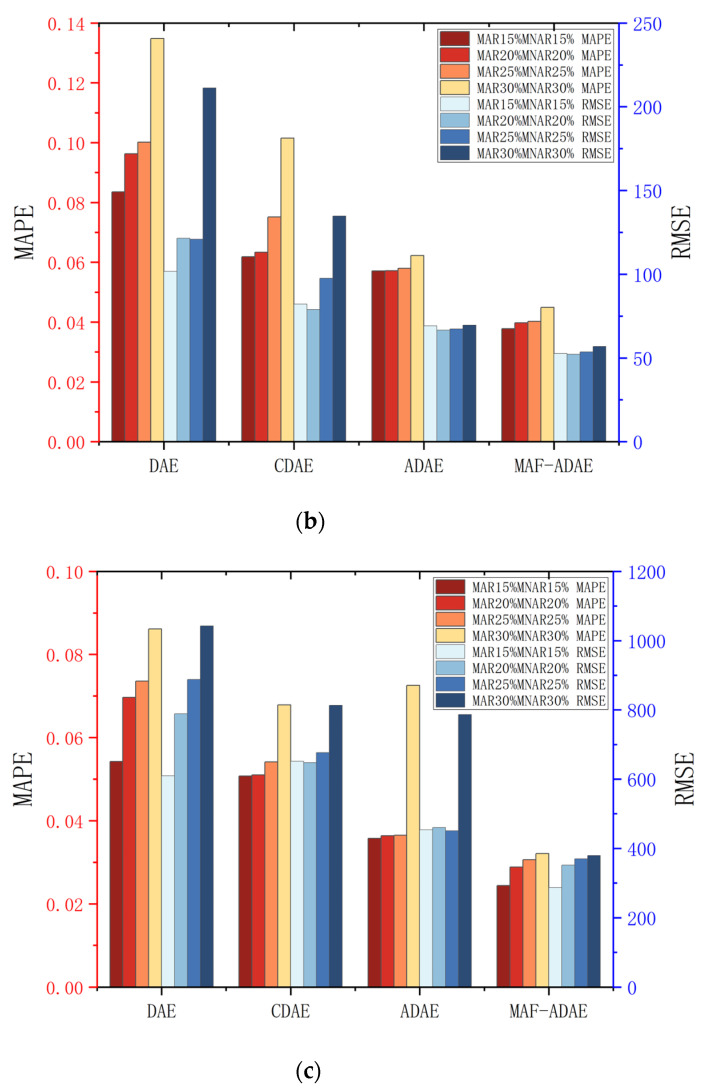
The SD of MAPE and RMSE in each imputation case. (**a**) Dataset C. (**b**) Dataset N. (**c**) Dataset A.

The MAF-ADAE is an improved autoencoder including ADAE and MAF. To verify their effectiveness and explain exactly what modules work for, we implemented the ablation experiments on multiple DAE-based models. Specifically, the DAE is composed of fully connected layers for both the encoder and the decoder, the CDAE network with the encoder as convolution and the decoder as deconvolution, and the ADAE without MAF. They are all tested in three datasets with multiple missing rates to demonstrate the effectiveness of the encoder, decoder, and filtering modules proposed in the MAF-ADAE. The MAPE and RMSE of the models in the C, E, and A datasets are shown in Figure 8a–c.

Figure 8a–c demonstrate that the performance and stability of the MAF-ADAE are consistent and excellent in the three experimental cases. For example, compared to the DAE and CDAE, the ADAE can effectively improve the imputation accuracy for the majority of missing data. The ADAE benefits from the asymmetric structure with skip connections and improved loss function, and can extract features and reconstruct data more efficiently. Once the data has a high missing rate, its imputation accuracy will be seriously reduced, which is especially clear in Figure 8c. At this time, the MAF can compensate for the imputation error under a heavy and extreme missing scenario by the continuity of the data. Not only that, it can also slightly improve the accuracy of the low-missing-rate data imputation.

## 5. Conclusions

In this paper, we developed a customized end-to-end model to address the multi-type missing imputation of power equipment monitoring data. As the improper handling of missing data can have serious consequences, the proposed model aims to impute multi-type missing (i.e., MAR and MNAR) with different missing rates. This model is composed of two modules, namely ADAE and MAF. The ADAE completes the preliminary imputation of the missing data and the MAF further optimizes the imputed data. To demonstrate the efficacy of the proposed model, a series of simulations were conducted on the three time-series power equipment monitoring datasets. The results show the superior data imputation performance and stability of the proposed model, even in heavy and extreme missing scenarios. In addition, the impact of each module on the model performance is further investigated, and the results of ablation experiments illustrated the superiority of the ADAE-MAF in autoencoding models.

## Figures and Tables

**Table 1:** Performance comparison (in MAPE/RMSE) for imputation tasks on datasets (C), (N), and (A).

Imputation Cases	LI	TRMF	LRTC-TNN	SDAE	VAE	MAF-ADAE
MAR15%MNAR15%, C	0.246/1134.53	0.259/798.44	0.071/310.94	0.142/370.16	0.23/581.78	0.080/**244.48**
MAR20%MNAR20%, C	0.29/1440.74	0.269/886.74	0.115/538.92	0.196/546.27	0.242/615.7	**0.088/265.16**
MAR25%MNAR25%, C	0.478 /3455	0.398/796.05	0.158/675.88	0.219/629.57	0.243/584.46	**0.094/267.52**
MAR30%MNAR30%, C	0.809/8528.04	0.487/1133.82	0.207/806.99	0.271/859.35	0.212/553.69	**0.101/276.05**
MAR15%MNAR15%, N	0.104/227.06	0.117/274.4	0.078/103.94	0.084/101.62	0.128/148.88	**0.038/52.59**
MAR20%MNAR20%, N	0.115/239.23	0.12/288	0.088/115	0.096/121.41	0.135/157.93	**0.04/52.21**
MAR25%MNAR25%, N	0.169/398.67	0.228/336.88	0.099/179.78	0.1/120.96	0.146/160.14	**0.040/53.55**
MAR30%MNAR30%, N	0.248/585.67	0.248/335.49	0.117/197.29	0.135/211.2	0.212/235.5	**0.045/56.91**
MAR15%MNAR15%, A	0.093/2883.07	0.086/2373.8	**0.021**/339.04	0.054/609.46	0.086/1067.05	0.023/**287.31**
MAR20%MNAR20%, A	0.129/2551.59	0.112/2485.79	0.029/383.65	0.07/788.45	0.086/999.56	**0.029/351.39**
MAR25%MNAR25%, A	0.177/3120.04	0.132/2543.06	0.039/447.88	0.074/887.78	0.09/1034.15	**0.031/370.24**
MAR30%MNAR30%, A	0.212/3525.93	0.147/2630.68	0.04/536.02	0.086/1042.08	0.092/1052.86	**0.032/379.33**

**Table 2:** Performance comparison (in SD) for imputation tasks on datasets (C), (N), and (A).

Datasets	Models	MAR20%MNAR20%		MAR30%MNAR30%	
		SD of MAPE	SD of RMSE	SD of MAPE	SD of RMSE
C	LI	0.0518	362.111	0.0538	367.634
	TRMF	0.0318	68.942	0.0406	135.750
	LRTC-TNN	0.0096	53.569	0.0141	58.766
	SDAE	0.0228	43.951	0.0244	79.984
	VAE	0.0276	85.733	0.0345	79.721
	MAF-ADAE	**0.0054**	**30.716**	**0.0120**	**41.269**
N	LI	0.0078	41.278	0.0235	166.059
	TRMF	0.0046	28.955	0.0177	54.697
	LRTC-TNN	0.0084	24.471	0.0227	69.323
	SDAE	0.0062	9.804	0.0093	48.262
	VAE	0.0047	11.779	0.0130	12.231
	MAF-ADAE	**0.0014**	**5.162**	**0.0019**	**7.034**
A	LI	0.0065	120.037	0.0248	450.984
	TRMF	0.0051	49.448	0.0219	175.664
	LRTC-TNN	**0.0008**	34.595	0.0025	45.020
	SDAE	0.0028	44.113	0.0083	99.060
	VAE	0.0023	51.724	0.0109	68.564
	MAF-ADAE	0.0019	**22.659**	**0.0018**	**38.285**

## Data Availability

Data are contained within the article.
